# Cognitive Benefits of Exercise: Is There a Time-of-Day Effect?

**DOI:** 10.3390/healthcare10091766

**Published:** 2022-09-14

**Authors:** Reinaldo Maeneja, Inês S. Ferreira, Cláudia R. Silva, Ana Maria Abreu

**Affiliations:** 1 Institute of Health Sciences, Universidade Católica Portuguesa, Palma de Cima, 1649-023 Lisbon, Portugal; 2 Faculdade de Ciências da Saúde e Desporto, Universidade Save, Maxixe 1301, Mozambique; 3 Faculty of Social Sciences and Technology, Universidade Europeia, 1500-210 Lisbon, Portugal; 4 Center for Research in Neuropsychology and Cognitive and Behavioral Intervention (CINEICC), Faculdade de Psicologia e de Ciências da Educação (FPCE), Universidade de Coimbra, 3000-115 Coimbra, Portugal; 5 Psychological Assessment and Psychometrics Laboratory (PsyAssessmentLab), Faculdade de Psicologia e de Ciências da Educação (FPCE), Universidade de Coimbra, 3000-115 Coimbra, Portugal; 6 Escola Superior de Saúde de Alcoitão, 2649-506 Alcoitão, Portugal; 7 Center for Interdisciplinary Research in Health, Universidade Católica Portuguesa, 1300-477 Lisbon, Portugal

**Keywords:** physical activity, cognitive benefits, time of day

## Abstract

It is well established that physical activity benefits cognition. Further, the time of day one engages in physical activity has been suggested to influence cognition. Here, we aimed to understand if there is a time-of-day effect (morning or afternoon) of physical activity on cognition, i.e., if exercising in the morning or afternoon might bring greater cognitive benefits. A total of 56 participants were allocated to one of two groups with the same baseline cognitive performance as well as fitness level (International Physical Activity Questionnaire—IPAQ): 27 to the morning intervention (M) group; and 29 to the afternoon intervention (A) group. In both groups, the participants engaged in an intermittent recovery test (Yo-yo), 4 times a week for 12 weeks. All participants were assessed with the d2 Test of Attention and the Borg scale of perceived exertion pre- and post- acute and chronic intervention. After the first bout of exercise and after 12 weeks, we observed cognitive improvements both in the M and A groups. Surprisingly, we do not find differences between the time of day regarding cognitive benefits. Our results do not support the existence of a time-of-day effect for the attentional cognitive benefits of exercise.

## 1. Introduction

Knowledge about the benefits of physical exercise on cognition has been widely explored in recent years [1,2]. Physiological mechanisms of moderate exercise are associated with improvements in muscle function (power, strength, and endurance), motor skills (agility, balance, coordination, and speed), cardiorespiratory function (oxygen transport, heart and lung function, and blood pressure) and metabolic regulation (glucose tolerance, lipid, and lipoprotein metabolism) [3]. These mechanisms are likely to enhance brain function and cognitive ability through their integrative effect on circulation and cerebral activation [4]. Moreover, brain-derived neurotrophic factor (BDNF) secretion, catecholamines circulating in the bloodstream, and neurogenesis also impact cognition and are modulated by physical exercise [5]. From a psychological perspective, physical exercise has also been shown to impact mood, reduce symptoms of depression and anxiety [6], and increase the perception of subjective well-being [7]. At a social level, it allows social interaction and integration, particularly in the context of team sports [8]. Returning to the cognitive level, considerable research has shown that physical exercise enhances brain and cognitive outcomes across the life span, both in healthy adults [9] and in people with cognitive dysfunction [10]. Despite the heterogeneity of methodologies across studies (e.g., study design, exercise protocol, cognitive instruments used), the consistency of effects and effect sizes across populations affords the conclusion that physical exercise positively influences cognitive function [2]. This evidence has been associated with improvements in a wide range of cognitive test results, namely those measuring attention, processing speed, executive function, and memory [2,9,11,12]. Attentional processes underlie the efficiency of several cognitive domains (e.g., memory and executive-control processes), thus being crucial for global cognitive functioning. Selective attention entails processing parts of the sensory input to the exclusion of others, while sustained attention entails maintaining sensitivity to incoming stimuli for some time [13]. Both are involved in a variety of daily living activities and their optimization represents a potential cognitive and functional increment. The literature indicates that acute physical exercise can improve attentional processes, in particular selective attention [14,15,16]. Interestingly, watching movies involving human motor action was also associated with better performance in a selective attention task [17], results that could be amplified with effective physical exercise. 

The specific time of day one engages in physical exercise may also putatively influence cognitive performance. Previous literature points out a time of day effect on training response [18] and peak psychomotor performances in the afternoon [19]. Specifically, both physical and cognitive performances are influenced by the circadian rhythm due to the physiological variations occurring across the day mediated by the oscillation of hormone segregation in the body, e.g., [20,21]. However, the studies reporting on cognitive benefits of exercise in the morning and afternoon, are inconsistent. A recent study has found that exercise in the morning can increase metabolism, while exercise later in the day increases the amount of energy used due to the natural circadian rhythm of the body [22]. An increase in metabolism is consistent with an increase in cognitive performance. Others have found that a bout of moderate-intensity exercise in the morning can increase cognitive performance, by improving serum BDNF in older adults [23]. However, no afternoon condition was used in this study, and it remains unclear if cognitive performance would prove similar, after moderate-intensity exercise in the afternoon. Although there have been many accounts of cross-culture success tales concerning the benefits of morning exercise for cognition in the school context [5], to our knowledge, the studies examining the best time of day to perform physical exercise, to improve cognitive outcomes, are sparse and very heterogeneous. Indeed, a recent study [24] showed that acute exercise in the morning might hinder the retrieval of stored information and that the cognitive benefits of acute exercise are short-lived. These authors analyzed the retrieval of information encoded pre-exercise and pre-sudoku puzzle-solving and observed that participants in the exercise group showed decreased retrieval performance 8 h post-exercise while the sudoku puzzle-solving group maintained performance after 2, 5, and 8 h post-puzzle solving. Further, a recent study showed that a combined physical and cognitive exercise program seemed to be more efficient in improving cognitive functions in the afternoon [25]. It is not clear if the greater benefits were a consequence of the dual task protocol. While there is some evidence for morning benefits, it is scarce and inconsistent. Here we aim to understand if there is a time-of-day effect of physical exercise on cognitive performance. More specifically, we put forth the hypothesis that exercising in the morning should benefit cognitive functioning, namely, attentional performance. This is a groundbreaking study because it aims to shed new light on how time-tailored exercise might benefit cognition.

## 2. Materials and Methods

### 2.1. Participants

Since we aimed to identify what time of day (morning or afternoon) better improves cognition because of physical exercise, we carried out a between-subjects design wherein the participants were students and staff from a University in Mozambique recruited via announcements posted across the University Campus and following a snowball sampling strategy. 

The sample size was calculated based on the Facer protocol [26]. We calculated an a priori power analysis, using G*Power 3 software (Düsseldorf, Germany) [27] using F tests family and the corresponding approaches (ANOVA repeated measures). With respect to the input parameters, we specified a medium effect size of d = 0.04 [28,29], a significance level of α = 0.05 and a minimum power value of 80% (see, e.g., [30]). A sample size of 46 participants was estimated to have 90.0% power to detect an effect of the condition. However, since attrition is common in experimental research and to ensure a sample that responds to the elements considered in the sample calculation, we strived to recruit more than 60 participants. We were able to recruit 68 participants. Participants with resting blood pressure greater than 120/80 were excluded, as they were considered to be pre-hypertension [31,32]. Accordingly, two participants were excluded because they had a resting blood pressure of 143/70 mmHg. Further, participants who did not sign the informed consent, who later refused to participate, or who did not complete the protocol were withdrawn from the study. Four gave up the right to participate in the study because they did not return the signed consent, while six dropped out because, although they signed the consent, they never appeared in the sessions. Thus, from the initial 68 recruited participants, 56 were included in the final study. These 56 participants were aged between 18 and 45 years (23.67 ± 4.93). The participants were randomly allocated to the morning intervention (M) group (*n* = 27), and the afternoon intervention (A) group (*n* = 29). All participants were physically active, as they met the World Health Organization parameters of 75 min/week of vigorous physical activity or 150 min/week of moderate physical activity [33,34].

The study was approved by the Ethics Committee for Health of the Universidade Católica Portuguesa (CES-UCP) and by the National Committee on Bioethics for Health—Mozambique (CNBS). All participants provided written informed consent, and the study followed the ethical principles of the Declaration of Helsinki for research involving human subjects [35].

### 2.2. Study Design

**Figure 1:**
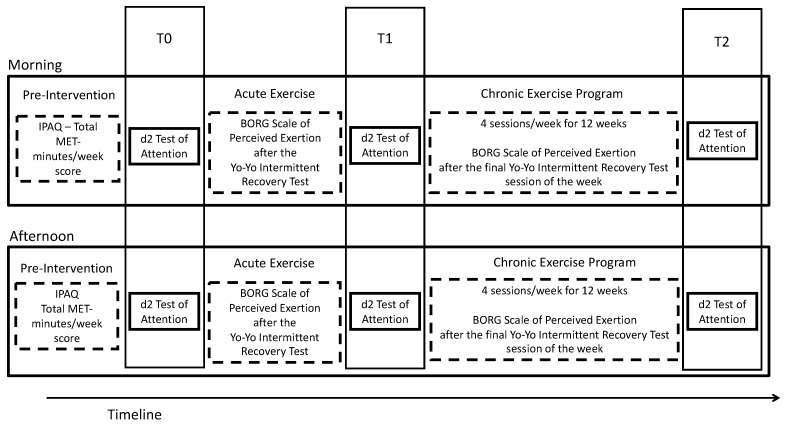
Research design timeline.

After medical evaluation and informed consent, 56 participants were randomly assigned to one of the two groups M or A. We acquired demographic information concerning the level of physical activity from all participants, for which we used the short form of the International Physical Activity Questionnaire (IPAQ) [36,37] to match the participants between groups. Since we aimed to investigate the putative differences between the potential cognitive benefits of physical exercise in the morning and afternoon, we developed a between-subjects design, wherein we had two intervention groups, without a control group, with the participants being randomly assigned to each group (M and A) to ensure that baseline characteristics were comparable across groups. Both groups of participants engaged in acute and chronic exercise (Yo-yo Intermittent Recovery Test) [38] similar to previous protocols [39] throughout 12 weeks. We acquired information concerning baseline, post-acute, and post-chronic exercise, and cognitive scores, using the d2 attention test [40]. We also acquired information concerning the perception of physical exertion through the Borg Scale [41] after the acute bout of exercise and after each week’s last session of exercise throughout the chronic 12-week exercise protocol, with 4 exercise sessions (Yo-yo test) per week. The M group performed the acute and chronic exercise sessions from 9:00 am to 12:00 pm, and A group performed the acute and chronic exercise sessions from 2:00 pm to 5:00 pm (see Figure 1).

### 2.3. Measures

In this study, we used the International Physical Activity Questionnaire (IPAQ), the Borg scale, and the d2 Test of Attention.

#### 2.3.1. The International Physical Activity Questionnaire 

The level of physical activity of each participant was assessed using the short form of the IPAQ [36,37]. This Questionnaire was developed to evaluate the weekly time spent on physical activities. The short form comprises 7 questions and takes about 10 min to complete [42]. This instrument was used before the beginning of the physical activity sessions to pair the participants regarding physical activity and to make sure that the exercise protocol was added to two groups with the same physical activity baseline. Scalar or parametric data can be obtained with the IPAQ. We opted for parametric data (METs/min per week) to match the participants. A MET is the ratio of the working metabolic rate relative to the resting metabolic rate. According to the American Heart Association, at least 500 METs/min a week are recommended for a healthy lifestyle, which is equivalent to 75 min/week of vigorous physical activity or 150 min/week of moderate physical activity [33,34].

#### 2.3.2. The Borg Scale

We monitored the participants’ perceived exertion during physical activity using the Borg subjective exertion scale, with scores ranging from 6 to 20 [43,44]. The Borg Scale or Borg Rating of Perceived Exertion (RPE) is a method to obtain subjective estimates of exercise effort and intensity both in healthy people and in patient populations [45]. Perceived exertion is defined as the ability to detect and respond to sensations arising from the body during exercise [46,47,48]. Thus, we used the Borg-RPE Scale at the end of the acute physical exercise session and at the end of each of the last weekly sessions along the 12-week chronic intervention protocol, to quantify the subjective feelings of fatigue and exercise tolerance that each participant underwent. Since physical fatigue seems to be task-specific and to negatively affect performance in memory-demanding tasks or have no effect in target detection tasks [49], we were interested in understanding how the perception of exertion might affect the different performance indices of the d2 test of attention. Moreover, previous work has shown that fatigue seems to specifically affect the more difficult tasks, and this is suggested to be related to arousal and the allocation of processing resources [50].

#### 2.3.3. The d2 Test of Attention

The d2 Test of Attention [40] is a paper-and-pencil cancellation test to measure attention, visual scanning, and processing speed, that involves the presentation of visual stimuli, i.e., 14 lines of 47 “p” and “d” characters adjacent to each other. The d2 Test of Attention has been proposed as a useful form of measuring two processes, attention, and concentration, that have been directly associated with the cognitive functions of our brain [51]. Overall test duration is 4 min 40 s. The number of correct responses is regarded as a measure of concentration performance, indicative of selective attention and sustained attention, while the number of commission and omission errors is seen as a measure of control, sustained attention, inhibitory control, and impulsivity [52,53]. This measure allows the collection of several performance indices. Of interest to us was: the General Efficiency score, consisting of the total number of characters processed minus the total errors (TC-E) and representing an indicator of attention control and the relationship between speed and accuracy in the task; the Concentration Index (CI) representing the total hits minus the total omission errors is an excellent measure of concentration ability allowing the assessment of the combination between speed and precision; the Error Percentage (E%), represents the percentage of total errors, i.e., commission errors (related to inhibitory control) + omission errors (related to attention control) made during the test and affords the assessment of the qualitative aspects of performance; and the Variability Index (VI), representing the difference between the maximum and the minimum number of processed characters, is an indicator of consistency and stability in task performance, i.e., temporal persistency across the task [52]. 

We chose this test because it assesses attention and concentration and physical activity has been associated with beneficial effects on attention/concentration capacity [54,55]. The d2 Test of Attention was assessed before the physical activity session (Yo-yo Intermittent recovery test) and immediately after the acute intervention and 12 weeks later, after the final session of the intervention protocol.

### 2.4. Procedure

We chose to apply the Yo-yo Intermittent Recovery Test (Yo-yo IRT) [38] as our exercise protocol. It consists of repeated exercise bouts performed at progressively increasing speeds, interspersed with 10 s rest periods. In the Yo-yo IRT, participants perform repeated 2 × 20 m runs at progressively increasing speed, interrupted by 10 s recovery periods. The test is carried out until the participant is completely exhausted. The rhythm is controlled by an acoustic device, indicating start, turn and end. The test can be discontinued if the participant fails twice to reach the finish line in time or discontinues the test [38,56,57]. Depending on conditioning, the Yo-yo IRT can be extended from 5 to 20 min [38,57,58]. This test is widely used to assess cardiorespiratory fitness. However, this was not our aim. We chose this test because it is easy to use, it assesses an individual’s ability to repeat performance intermittently over a long period, allowing it to be used by anyone regardless of training [38,58,59,60,61]. Moreover, the higher the fitness level, the lower the fatigue [62]. In this study, each session had a maximum duration of 20 min. Each participant gained cardiovascular fitness since in level 1 of the Yo-yo IRT, exhaustion is reached between 10 and 20 min. 

Physiological measures associated with the use of this test show a surge in blood circulation and improvement in brain oxygenation [63,64,65,66,67], which increases cognitive benefits [68,69,70]. The Yo-yo IRT was preceded by a 5-min warm-up period. Three research assistants who were blind to the aim of the study supervised the exercise sessions. 

To characterize the sample, we used inferential statistics to verify if there were significant differences between the two groups (M vs. A) concerning gender, age, and METs-minute/week (IPAQ Scale) (Table 1). We computed a Chi-Square test that showed no significant differences between the 2 groups. Both the M and A groups, present a higher percentage of female subjects (M = 55.6%, A = 69%; *p* = 0.300). Regarding age, we computed a *t*-test that did not reveal any significant differences (*p* = 0.154) between the two groups. In the M group, the ages ranged between 18 and 32 years, (M = 23.37 years) and in the A group, the ages ranged between 17 and 33 years (M = 21.93). Moreover, we did not find any significant differences between the two groups concerning METs-minute/week (M = 1460.13, A = 1228.00; *p* = 0.112).

Normality was verified using the Shapiro–Wilk test. Although normality was not found in all the variables in the two groups (M and A), only minor deviations were found, and the Skewness and Kurtosis values were lower than 2 and 7, respectively, which afforded the use of parametric statistics [71]. Further, we computed a repeated-measures ANOVA to assess intra-group differences and the Least Significant Difference (LSD) post hoc test to verify, within each group, the existence of significant differences between the 3 evaluation moments (T0, T1, and T2) in the different cognitive dependent variables of the study (i.e., the analyzed d2 performance indices: General Efficiency (TR − (O + C)); Concentration Index (TA-C); Variation Index (TR+ − TR-); and Error Percentage (E% = (100(O + C))/TR).

## 3. Results

### 3.1. General Efficiency

**Figure 2:**
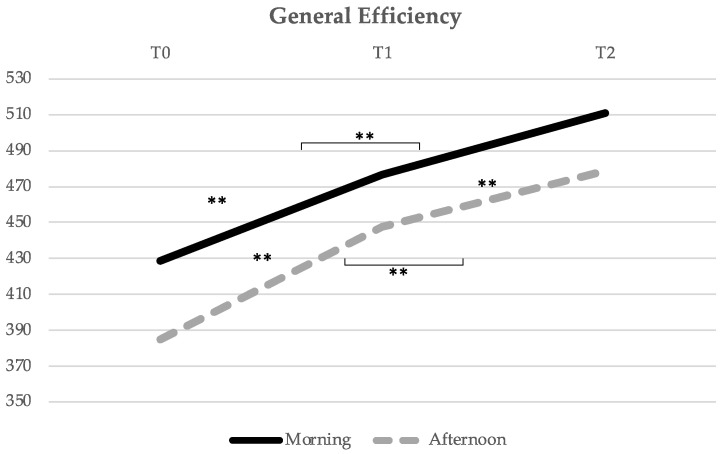
Evolution of the General Efficiency mean score over time (from T0 to T1, i.e., from pre- to post-acute exercise; and from T1 to T2, i.e., from post-acute exercise to post-chronic exercise). ** Indicates significant differences for *p* < 0.01.

Concerning the General Efficiency performance index (Figure 2), the M group had a significant evolution of the General Efficiency mean score over time (F = 7.874, *p* = 0.001), with a significant increase from T0 to T1 (*p* = 0.034) and from T0 to T2 (*p* = 0.000), with no significant difference between T1 and T2 (*p* = 0.114). In the A group, there was a consistent significant increase between different times (F = 18.891, *p* = 0.000), from T0 to T1 (*p* = 0.002), from T0 to T2 (0.000), and from T1 to T2 (*p* = 0.033). 

### 3.2. Concentration Index

**Figure 3:**
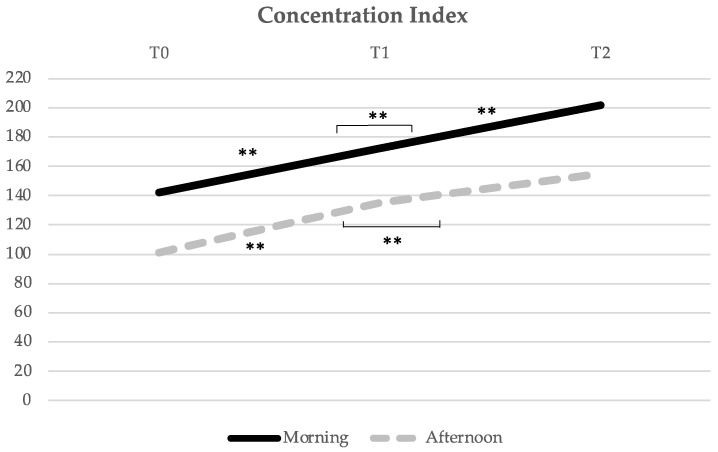
Evolution of the Concentration Index mean score over time (from T0 to T1, i.e., from pre- to post-acute exercise; and from T1 to T2, i.e., from post-acute exercise to post-chronic exercise). ** Indicates significant differences for *p* < 0.01.

Concerning the Concentration Index (Figure 3), the M group also had a significant evolution of the Concentration Index mean score over time (F = 9.639, *p* = 0.000), with a significant increase between all evaluation moments, from T0 to T1 (*p* = 0.041), T0 to T2 (*p* = 0.001), and T1 to T2 (*p* = 0.006). In the A group, there was also a significant increase over time (F = 12.591, *p* = 0.000), from T0 to T1 (*p* = 0.014) and from T0 to T2 (0.000), but not between T1 and T2 (*p* = 0.060). 

### 3.3. Error Percentage

**Figure 4:**
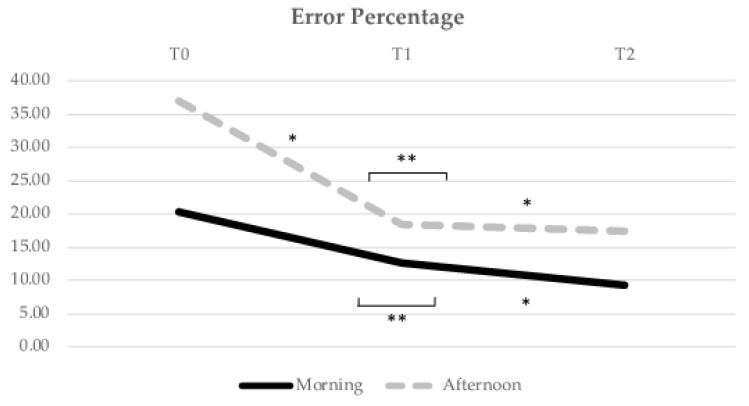
Evolution of the Error Percentage mean score over time (from T0 to T1, i.e., from pre- to post-acute exercise; and from T1 to T2, i.e., from post-acute exercise to post-chronic exercise). * Indicates significant differences for *p* < 0.05; ** Indicates significant differences for *p* < 0.01.

Concerning the Error Percentage performance index (Figure 4), the M group had a significant decrease in the mean Error Percentage score over time (F = 5.295, *p* = 0.022). No significant decrease was found in the significance threshold from T0 to T1 (*p* = 0.080). A significant decrease was found from T0 to T2 (*p* = 0.010), and a significant difference from T1 to T2 (*p* = 0.044). In the A group, there was also a significant evolution of the mean Error Percentage score over time (F = 3.568, *p* = 0.02). There was a significant decrease from T0 to T1 (*p* = 0.03), a significant decrease from T0 to T2 (*p* = 0.042), and no significant difference from T1 to T2 (*p* = 0.604). 

### 3.4. Variation Index

**Figure 5:**
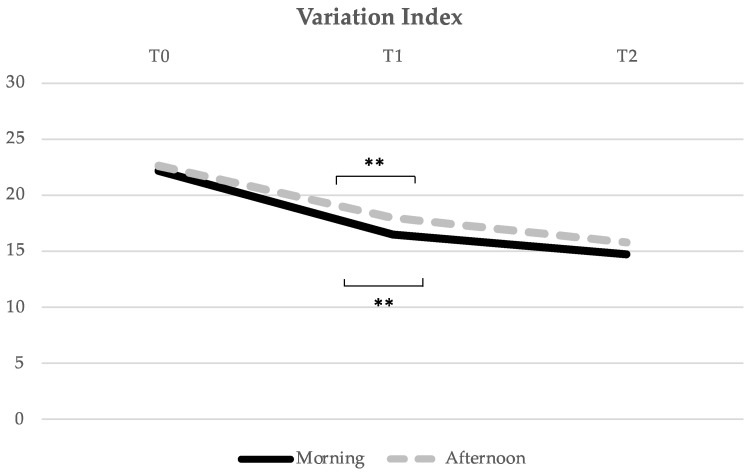
Evolution of the Variation Index mean score over time (from T0 to T1, i.e., from pre- to post-acute exercise; and from T1 to T2, i.e., from post-acute exercise to post-chronic exercise). ** Indicates significant differences for *p* < 0.01.

In the Variation Index (Figure 5), the M group had a significant evolution of the Variation Index mean score over time (F = 3.484, *p* = 0.038), with no significance threshold from T0 to T1 (*p* = 0.069), a significant decrease from T0 to T2 (*p* = 0.016), and no significant difference from T1 to T2 (*p* = 0.576). In the A group, the results were identical. There was also a decrease in the Variation Index mean score over time (F = 5.506, *p* = 0.007). From T0 to T1, there was no difference in the significance threshold (*p* = 0.057), from T0 to T2 there was a significant difference (*p* = 0.001), and from T1 to T2, no significant difference was found (*p* = 0.290).

As described above, in the intra-group analysis, there is a significant evolution over time in both groups in all d2 performance indices. Further, for each performance index, we computed the relative differences, by assessing the difference between the mean scores at the different time points (T1-T0; T2-T1; and T2-T0) thus obtaining a new score concerning the relative improvement between two testing times [17,33]. The rationale for this is that we assume that we should find an improvement between the two testing times simply due to the repetition and familiarization with the cognitive test. Hence, we aim to verify if these relative improvements are more expressed in the M or A group. We computed a *t*-test for independent samples to compare the two groups in the relative improvements occurring over time. The *t*-test did not reveal any significant differences between the M and the A groups in the changes observed over time in the different performance indices (Table 2).

**Figure 6:**
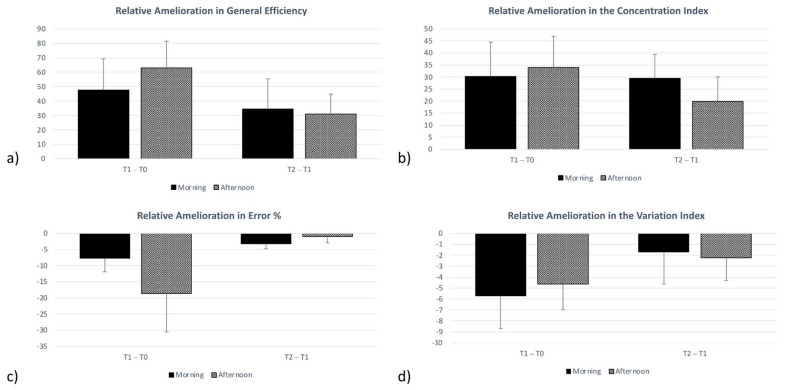
Relative Amelioration Scores in (**a**) General Efficiency; (**b**) Concentration Index; (**c**) Error Percentage; and (**d**) Variation Index. Error bars represent Standard Errors. No significant differences were found.

Figure 6 showcases the absence of differences between the M and A groups across the different time points (from T0 to T1 and from T1 to T2).

**Figure 7:**
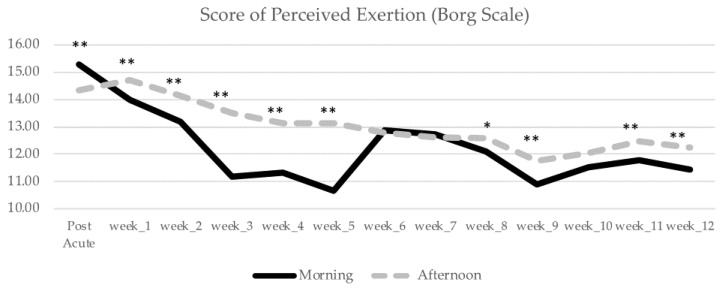
Score of perceived exertion across different time-points (post-Acute T1 and at the end of the last session of each week of the 12-week intervention program, including T2, the final session) for the M and A groups. * Indicates significant differences between M and A for *p* < 0.05; ** Indicate significant differences between M and A for *p* < 0.01.

Furthermore, we computed Pearson Correlations between the score of perceived exertion given by the Borg scale Post-Acute (T1) and the performance indices at the same time point, as well as between the performance indices at Post-Chronic (T2) and the score of perceived exertion given by the Borg scale at the same time point. None of these correlations yielded significance (all *p* > 0.05). In Figure 7 we showcase the progress of the score of perceived exertion across the different time points.

An independent samples *t*-test did not reveal any significant differences between M and A in week 6 [M (M = 12.87, SD = 0.99) and A (M = 12.79, SD = 1.18), *t*(54) = 0.274, *p* = 0.785]; in week 7 [M (M = 12.73, SD = 1.07) and A (M = 12.62, SD = 0.85), *t*(54) = 0.420, *p* = 0.676]; or in week 10 [M (M = 11.53, SD = 1.07) and A (M = 12.03, SD = 1.36), *t*(54) = −1.541, *p* = 0.136]. In the remaining weeks, the A group reveals a greater perceived exertion (*p* < 0.05). 

## 4. Discussion

The present study aimed to assess if there is a time-of-day effect on the cognitive benefits of physical exercise, i.e., if it is better to perform physical exercise in the morning or in the afternoon to obtain the greatest cognitive gains. Although the literature is scant and heterogeneous it seemed to point out to greater benefits of physical exercise in the morning. Thus, with this study, we aimed to verify this. We constituted two groups according to the time of day of physical exercise (morning—M or afternoon—A), who were equivalent in sociodemographic characteristics (age, gender) and activity level (IPAQ), and assessed these groups using measures of attention (d2 Test of Attention) and perceived exertion (The Borg RPE) before and after acute exercise and after chronic exercise (Yo-yo Intermittent Recovery test). 

Our results highlight a relative improvement in selective and sustained attention outcomes after both acute and chronic exercise, with no significant influence of the time of day in which it was performed. More specifically, results concerning general efficiency and concentration ability achieved a significant improvement after acute (T0 to T1) and chronic (T0 to T2) exercise, with no significant differences between the M and A exercise groups. Regarding the qualitative aspects of attentional performance, the proportion of errors decreased over time in both groups, namely between T0 and T2, which supports an increase in the accuracy, meticulousness, and quality of attentional performance with the practice of chronic exercise. The stability and consistency of performance on the attentional task were equivalent between groups over time. These empirical data support the evidence that physical exercise improves attentional performance and cognitive functioning in young adults [14,15,16]. A justification for these results may be that the practice of moderate exercise over time is associated with cardiovascular regulation which increases blood circulation, and improves brain oxygenation [72], resulting in cognitive benefits [4]. Since attentional processes underlie the efficiency of several cognitive abilities, its improvement over time may also optimize overall cognitive functioning [2]. Such potential benefits were also reported in previous studies with healthy adults [9] and people with cognitive dysfunction [10]. Several clinical groups such as people with neurocognitive, neuropsychiatric, or neurodevelopmental disorders present attentional deficits and thus their cognition and activities of daily living may benefit from continuous exercise [10,73].

It is important to emphasize that physical exercise should be moderate [9], since high-intensity exercise may depress cognitive abilities [74,75]. Our results obtained from the Borg scale, a measure of perceived exertion and dyspnea associated with exercise, constitute an indirect measure of exercise intensity, which was perceived in average terms by both groups as having a light to hard intensity, and not being very hard. This indicator suggests the moderate nature of the exercise performed by the participants, which was applied to homogeneous groups in terms of physical activity. Possibly, the moderate nature of the exercise may have determined the improvement of attentional performance over time. It is possible that the participants improved their fitness level as they continued to exercise across the 12-week protocol. Since the higher the fitness level, the lower the fatigue [62], it is possible that an increase in fitness level contributed to the decrease in the perception of exertion. Even though only at weeks 6, 7, and 10 do the groups M and A express the same level of exertion, given the predicted continuous decrease in perceived exertion, it is possible that, if the protocol were prolonged, the groups might cease to differ, as the participants tend to the same fitness level. Unexpectedly, the perception of perceived exertion is not associated with an increase in cognitive benefits as would be predicted by the cardiovascular regulation theory associated both with fitness and cognitive function. It is possible that another confounding variable might differently mediate fitness and cognitive function. 

A study with handball goalkeepers investigated the goalkeepers’ cognitive performances across the day. The study showed that selective and constant attention was better in the morning [76] suggesting that cognitive activities in an athlete’s life should be scheduled in the morning. Although we do find both acute and chronic benefits for cognition of physical exercise practice, surprisingly, we did not find a time-of-day effect for these benefits. It is extremely important to further research in this area with large-sample systematic randomized controlled trials to support these findings. Crucially, and although we did not find a time-of-day effect of exercise on cognition, such a finding supports the possibility of more malleable training programs, appropriate for modern-day busy lives. 

Finally, this study is not without limitations. The study design did not include a control group, which would strengthen our results and conclusions. Although the morning and afternoon groups were homogeneous in terms of activity level and started at the same baseline in terms of performance in the cognitive task (d2 attention test) and fitness level (IPAQ) our results do not completely clarify whether groups with no activity level might show significant differences in cognitive performance over time. Thus, it is unclear if the cognitive benefits observed are due to the passage of time or due to physical exercise. Furthermore, self-reported data were also used to assess activity level (IPAQ) and perceived exertion (Borg scale), which may suffer from eventual biases (e.g., from memory, critical judgment, social desirability).

## 5. Conclusions

Here we show that the practice of acute and chronic exercise is associated with incremental outcomes in selective and sustained attention, without influence of the time of day at which it was performed. Moreover, we show that perception of exertion decreases with continued chronic exercise suggesting that an increase in physical fitness should be associated with decrease in fatigue. However, we did not find an association between cognitive benefits and perception of exertion. These data show that a tailored exercise prescription does not benefit cognition according to the time of day one exercises. Since attention is a fundamental basic process in cognitive functioning and in achieving success in more complex tasks, the practice of moderate exercise over time may lead to improvements in cognitive functioning and in activities of daily living independently of the time-of-day it is practiced. Further studies are recommended to clarify and deepen the impact of time of day of different and specific exercises (type, frequency, duration, intensity) on cognitive functioning, immediately and over time, using a general and broad cognitive assessment, considering different age groups and populations, and investigating other possible confounding variables.

## Figures and Tables

**Table 1:** Participant characteristics and demographics.

		Group				
		Morning (*n* = 27)		Afternoon (*n* = 29)		
		Freq	%	Freq	%	
**Gender**	**Female**	15	55.6%	20	69.0%	X2 = 1.073 *p* = 0.300
	**Male**	12	44.4%	9	31.0%	
**Age**		18–32 23.37 ± 4.09		17–33 21.93 ± 3.33		*t* = 1.446 *p* = 0.154
**IPAQ** **MET_minWeek**		340.00–2754.00 1460.13 ± 493.48		325.00–2807.50 1228.00 ± 602.33		*t* = 1.571 *p* = 0.122

No significant differences were found.

**Table 2:** Independent samples *t*-test: Comparison of the 2 groups (M vs. A) in the Relative Amelioration Scores (T1-T0; T2-T1; T2-T0).

	Group	*n*	Mean	Std. Deviation	Independent Sample *t*-Test
Gen_PerformT1-T0	Morning	27	47.74	110.95	*t* = −0.546 *p* = 0.587
	Afternoon	29	63.07	99.05	
Gen_PerformT2-T1	Morning	27	34.33	109.07	*t* = 0.137 *p* = 0.891
	Afternoon	29	30.93	74.07	
Gen_PerformT2-T0	Morning	27	82.07	103.69	*t* = −0.492 *p* = 0.625
	Afternoon	29	94.00	76.43	
Conc_IndexT1-T0	Morning	27	30.30	73.38	*t* = −0.190 *p* = 0.850
	Afternoon	29	33.93	69.90	
Conc_IndexT2-T1	Morning	27	29.56	51.31	*t* = −0.681 *p* = 0.499
	Afternoon	29	19.90	54.61	
Conc_IndexT2-T0	Morning	27	59.85	83.88	*t* = 0.326 *p* = 0.746
	Afternoon	29	53.83	48.68	
Erro%_T1-T0	Morning	27	−7.67	21.88	*t* = 0.839 *p* = 0.405
	Afternoon	29	−18.60	64.29	
Error%_T2-T1	Morning	27	−3.26	8.00	*t* = −0.904 *p* = 0.370
	Afternoon	29	−1.01	10.37	
Error%_T2-T0	Morning	27	−10.93	20.51	*t* = 0.679 *p* = 0.500
	Afternoon	29	−19.61	63.41	
Variation_IndexT1-T0	Morning	27	−5.70	15.62	*t* = −0.277 *p* = 0.783
	Afternoon	29	−4.66	12.64	
Variation_IndexT2-T1	Morning	27	−1.70	15.27	*t* = 0.151 *p* = 0.880
	Afternoon	29	−2.24	11.18	
Variation_IndexT2-T0	Morning	27	−7.41	14.93	*t* = −0.150 *p* = 0.881
	Afternoon	29	−6.90	10.31	

## Data Availability

The data that support the findings of this study are available from the corresponding author upon reasonable request.
